# Editorial: Culture and second language (L2) learning in migrants

**DOI:** 10.3389/fpsyg.2022.968279

**Published:** 2022-07-15

**Authors:** Adrian Pasquarella, Fanli Jia, Aline Ferreira, Alexandra Gottardo, John W. Schwieter

**Affiliations:** ^1^School of Education, University of Delaware, Newark, DE, United States; ^2^Department of Psychology, Seton Hall University, South Orange, NJ, United States; ^3^Department of Spanish and Portuguese, University of California, Santa Barbara, CA, United States; ^4^Department of Psychology, Wilfrid Laurier University, Waterloo, ON, Canada; ^5^Department of Languages and Literatures, Wilfrid Laurier University, Waterloo, ON, Canada

**Keywords:** culture, second language learning (L2 learning), language and literacy development, environmental influences, immigration & migration, language acquisition, socio-cultural environmental differences, multilingual learners

Culture and language are synergistically intertwined. Culture is constructed by people, and often represented and communicated through oral and written forms of language. In turn, culture shapes the acquisition and development of cognition, knowledge, language, and literacy skills. By nature, language and literacy acquisition and development occur in a dynamic socio-cultural environment, with both proximal and distal influences that relate to individual differences in learning trajectories and outcomes. Variability across social-culture environments is vast and an individual's prior experiences and knowledge meaningfully influence outcomes in language and literacy acquisition.

Advances in transportation and communicative technology enhanced human capacity to experience and learn other languages. Increased globalization and migration solidified an interest and necessity for many people to learn a second language (L2). In reality, learning a L2 or additional language is a necessity for most people to thrive in their environment. Therefore, second language and literacy acquisition is an integral part of educational, political, and social discourse and decision making. However, more systematic, rigorous, and empirically driven research is needed to increase our understanding of the dynamic and synergistic nature between socio-cultural environments and L2 learning across the life-span. This Research Topic included four eclectic articles focused on investigating the relationship between culture and second language learning.

Liu et al. examined how bilingual language control adapts to the cultural context. The authors employed a carefully designed picture naming task that included culturally-neutral and culturally-biased pictures, as well as, culturally congruent and incongruent naming conditions. Results demonstrated asymmetries in adaptive processes and more uniform influences in proactive processes. The authors suggest that cultural context appears to play more of a role in modulating reactive but not proactive language control. The authors connect the dynamic nature of language control to important theoretical advancements.

Hao et al. examined similarities and differences in the written and oral syntactic networks of L2 learners. Writing corpora was compiled from the L2 learners in-class compositions, and the oral corpora was collected through a topic-based speaking test. The authors examination of neural network parameters of the interlanguage written and oral syntactic networks identified that all networks initially present scale-free and small-world properties. The authors discuss how these quantitative parameters relate to L2 acquisition and proficiency. Hao et al. conclude with a thoughtful discussion regarding the roles of innate language devices and first language knowledge on second language acquisition.

Uchikoshi et al. examined the relationship of parental reports of acculturation on children's bilingual language abilities. Bilingual children were assessed on oral language measures in both their L1 (Spanish or Chinese) and L2 (English). Interviews were conducted with parents to collect parental acculturation and self-reported language proficiency. Groups of Chinese American and Mexican American parents scored similarly on the majority of the acculturation dimensions. Cluster analysis produced four groups of children based on their bilingual ability: high language ability in both the L1 and L2, low language ability in both, L2-dominant, and L1-dominant. In relation to acculturation, the authors report more similarities than differences among the four groups with parents in all groups reporting stronger ties to their heritage culture and L1 than to the American culture. The authors discuss the interesting linkages between parental cultural identities and children's language dominance.

Ibáñez-Alfonso et al. examined the role of socioeconomic status and cultural background on understanding individual differences in reading comprehension with first language (L1) and L2 Spanish speaking students. Measures of socioeconomic status (SES), non-verbal, reading comprehension, knowledge of syntax, sentence comprehension monitoring, and vocabulary were collected. The authors reported that after controlling for SES, differences in reading comprehension were negligible between L1 and L2 students. However, L2 students demonstrated lower performance on oral language tasks. Furthermore, the authors reported that variability in Spanish reading comprehension was significantly predicted by age, socioeconomic status, non-verbal reasoning, and comprehension monitoring. The authors discuss an overshadowing role of SES on language and literacy outcomes for both L1 and L2 students. The authors conclude that irrespective of the language status (L1 vs. L2), children in economically impoverished settings are at greater risk of Spanish reading comprehension difficulties.

Unpacking and understanding the cognitive-linguistic systems implicated in L2 acquisition and how culture influences, imprints, and ultimately shapes outcomes for L2 learners is a monumentally important undertaking. The articles presented in this Research Topic demonstrated rigorous approaches aimed at articulating the nature of culture on second language acquisition. The process is undoubtedly complex. Identifying and tracking cascading influences from macro- to micro-level socio-cultural contexts is a necessary step in understanding second language acquisition across diverse environments and instructional settings. Research needs to move toward a comprehensive, holistic, and culturally responsive approach to understanding the strengths and learning needs of second language learners. Importantly, examining evidence-based practices and data-driven decision making in educational and political settings is needed as a focus to ensure L2 learners build knowledge and capacity to understand, communicate, participate, and flourish across diverse socio-cultural environments.

## Author contributions

AP wrote the editorial. FJ, AF, AG, and JS reviewed the editorial. All authors contributed to the article and approved the submitted version.

## Conflict of interest

The authors declare that the research was conducted in the absence of any commercial or financial relationships that could be construed as a potential conflict of interest.

## Publisher's note

All claims expressed in this article are solely those of the authors and do not necessarily represent those of their affiliated organizations, or those of the publisher, the editors and the reviewers. Any product that may be evaluated in this article, or claim that may be made by its manufacturer, is not guaranteed or endorsed by the publisher.

